# Reducing the burden of orthodontic care for children with clefts: evaluating the effectiveness of pre-alveolar bone graft orthodontics in unilateral non-syndromic cleft patients (PABO study)— A study protocol for a multicentric randomised controlled trial

**DOI:** 10.1186/s13063-021-05505-0

**Published:** 2021-08-28

**Authors:** Badri Thiruvenkatachari, Syed Altaf Hussain, Puneet Batra, Charanya Vijayakumar, Manoj Prathap. C

**Affiliations:** 1 Department of Orthodontics, Sree Balaji Dental College and Hospital, Bharath Institute of Higher Education and Research, Velachery Main Road, Chennai, Tamil Nadu 600100 India; 2 Plastic and Reconstructive Surgery, Sriramachandra Institute of Higher Education and Research, No.1, Ramachandra Nagar, Porur, Chennai, Tamil Nadu 600116 India; 3 Saint Parmanand Hospital, 18, Sham Nath Marg, Civil Lines, New Delhi, 110054 India; 4 Sree Balaji Dental College and Hospital, Bharath Institute of Higher Education and Research, Velachery Main Road, Chennai, Tamil Nadu 600100 India

**Keywords:** Pre alveolar bone graft, Alveolar bone graft. ABG, Pregraft orthodontics, Cleft palate, Cleft alveolus, SABG, Cleft orthodontics

## Abstract

**Background:**

An alveolar cleft commonly affects 75% of cleft lip and palate patients. While it is common practice to provide a course of orthodontic treatment before alveolar bone grafting, there are no previous high-quality studies reporting on the benefits of this type of treatment.

**Aim:**

The aim of the study is to evaluate the effectiveness of pre-alveolar bone graft orthodontics for unilateral non-syndromic cleft palate patients.

**Method:**

The PABO trial is a multicentric, parallel, two-arm, single-blinded randomised controlled trial. The inclusion criteria include unilateral cleft alveolus patients requiring bone graft and between the age group of 8 and 13 years with erupted upper central incisors. Participants will be recruited at three centres across India. Participants will be randomised to orthodontic treatment or no orthodontic treatment group. Both groups of participants will have alveolar bone graft surgery and will be followed up for 6 months after surgery. The primary outcome will be the success of the alveolar bone graft measured by anterior oblique radiograph and secondary outcomes include quality of life, cost analysis and quality of the dento-occlusal outcome. Data analysis will be carried out by an independent statistician at the end of the study.

**Discussion:**

This study is the first to evaluate the effect of orthodontics on alveolar bone graft success. The increased burden of care for these patients with multiple treatments required from multiple specialists from birth to adult life highlights the need for reducing unnecessary treatment provision.

**Trial Registration:**

Clinical Trials Registry – India, CTRI/2020/10/028756. Trial prospectively registered on 29 October 2020.

.

## Administrative information

**Table:** 

Title {1}	Reducing the burden of Orthodontic Care for children with Clefts: Evaluating the effectiveness of Pre-alveolar Bone Graft Orthodontics in unilateral non-syndromic Cleft patients (PABO Study)
Trial registration {2a and 2b}.	Clinical Trials Registry India. The trial has been submitted for registration (reference number: REF/2020/09/036904) CTRI is a Primary Register of the International Clinical Trials Registry Platform (ICTRP) (http://www.who.int/ictrp/search/en/), registered trials are freely searchable both from the WHO's search portal, the ICTRP as well as from the CTRI (www.ctri.nic.in)
Protocol version {3}	Protocol version 3.2 dated 25/12/2020
Funding {4}	Science and Engineering Research Board, Government of India
Author details {5a}	Badri Thiruvenkatachari (CI), Altaf Hussain, Puneet Batra, Manoj Prathap. C
Name and contact information for the trial sponsor {5b}	Bhuminathan. S, Registrar, Bharath Institute of Higher Education and Research 173 Agharam Road Selaiyur,Chennai - 600 073Tamil Nadu, India E-Mail: registrar@bharathuniv.ac.in Phone: 9444023359
Role of sponsor {5c}	Ensures ethics approval is obtained before commencement Ensures the trial runs according to good clinical practice Ensures trial is conducted in accordance with the protocol Notify appropriate authorities of any serious breach Appoint individuals for running the trial Keep a record of all adverse events (SAE, UE)

## Background

Cleft lip and palate (CLP) are among the most common congenital malformations with an overall incidence of around 1 in 700 individuals newborns [1], and as a result, between 27,000 and 33,000 children in India are born with clefts every year [2]. CLP is accompanied by a wide variety of dental and skeletal anomalies, which have a long-term impact on the patient’s facial aesthetics, function and self-esteem. CLP affects a range of anatomical areas, and the optimal management of a child with cleft demands an organised multidisciplinary effort involving otolaryngology, plastic surgery, maxillofacial surgery, orthodontics, speech and language therapy, audiology, paediatrics, nursing, genetics, psychology, and social work. Despite general progress in medical care, cleft lip and palate and related craniofacial anomalies have largely remained ‘orphan’ conditions, falling between several main disciplines. Treatment provision lacks a firm evidence base and most published research has been uncoordinated. The care for these children is marked by controversies with multiple protocols concerning treatment [3]. Additionally, the care for this group of children is being delivered in a fragmented way through several centres in India [2]. In a previous study looking at the burden of orofacial clefting (OFC), it has been reported that a total of 79,430 cleft patients had unmet treatment needs which accounted for 18.76% of the total Indian cleft population [4].

An alveolar cleft commonly affects 75% of cleft lip and palate patients [5]. The cleft may influence the position of the teeth and the dental arches, cause speech problems, exacerbate facial asymmetry, and result in a lack of bone support for the anterior teeth [6]. It is now standard practice to correct this problem by bone grafting the cleft alveolus before the eruption of the permanent canines [7]. It is also common clinical practice to provide a course of orthodontic treatment to correct any discrepancy between the alveolar segments and widen the cleft alveolus to improve surgical access. While it is common practice to provide a course of orthodontic treatment before alveolar bone grafting, a recent systematic review of the literature reveals that most studies underpinning this procedure are retrospective analyses of case series [5]. Importantly, there has been no prospective randomised trial on the benefits and relative costs of this procedure. This has led to some diversity of practice in India with some Indian centres carrying out pre bone graft orthodontic treatment routinely, while others do not.

## Rationale

A child undergoing pre-graft orthodontic treatment has to attend the Cleft Centre over a 6–15-month period, on amonthly basis and is also subject to the risks of orthodontic treatment, for example, dental decay, gingivitis, and root resorption [8]. It could be argued that this increases the burden of care for the child and parent and overall treatment costs, particularly if the treatment extends into the definitive phase of care. Furthermore, they have to undergo orthodontic treatment at an earlier age than their peers and may feel ‘set apart’ from their friends and classmates.

There is a serious lack of research in terms of comprehensive cleft care of UCLP patients at various stages of development in India. To our knowledge, there are no high-quality studies on pre alveolar bone graft orthodontics for cleft patients in India. Hence, this proposed study is aimed at evaluating the need for prealvoelar cleft orthodontics, reducing the burden and at the same time standardising and improving the quality of care for these patients.

## Methods

### Aim

To aim of this study is to compare the effectiveness of pre alveolar bone graft orthodontic treatment with no orthodontic treatment for children with unilateral cleft palate requiring alveolar bone graft surgery.

### Objectives

1. To evaluate the effectiveness of pre alveolar bone graft orthodontic treatment for children with unilateral cleft palate.

### Null hypothesis

The use of pre graft orthodontics is no more effective than no orthodontic treatment for cleft alveolus patients.

### Design

**Fig. 1:**
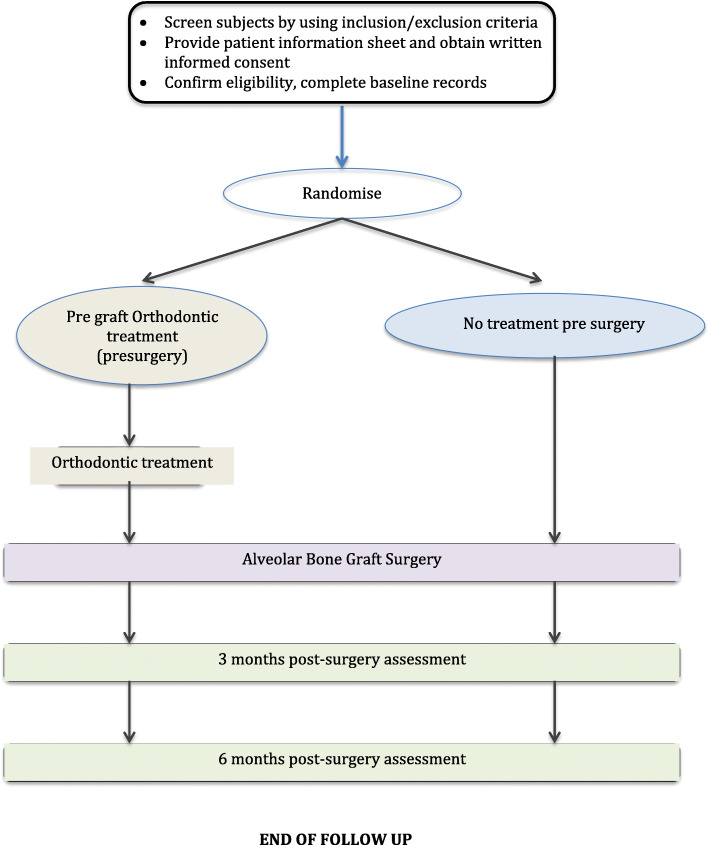
Schematics of trial design

Prealveolar bone graft Orthodontics (PABO) study is a 1:1 parallel group multicentre trial. Children aged between 8 and 13 will be randomised to either pregraft orthodontics group or no orthodontic treatment group. The study design is illustrated in Fig. 1.

### Study setting

The trial will be coordinated from Sree Balaji Dental College, Bharath Institute of Higher Education and Research, India. The trial will be conducted by cleft teams based in three centres across India. Criteria for the selection of sites are based on the previous experience in research and the volume of patients seen. The cleft team at each centre will include an orthodontist, cleft surgeon, paediatrician and cleft nurse.

### Eligibility criteria

Patients referred to cleft lip and palate centres will be included if they satisfy the below inclusion criteria:

- Unilateral cleft alveolus requiring alveolar bone graft
- Upper central incisors erupted.
- Aged 8 and 13 years old
- Written informed consent
- One care/parent must be a native language speaker of the state of residence.

Patients with any of the following will be excluded from the study:

- Bilateral cleft palate
- Patients with syndromes or other medical condition
- Subjects involved in any other orthodontic trials
- Inadequate oral hygiene

### Enrolment

The surgeon and orthodontist at each centre will assess the patient for inclusion and exclusion criteria. If the patient is identified as a potential patient, the parent/guardian will be invited, and information provided about the trial and the process of enrolment. The patient information sheet and consent form will be provided to the parent/legal guardian and they will then be given ample time to read and understand the forms and ask any questions. If the parent declines to participate, they will receive treatment as current practice and will receive the same level of support as patients participating in the trial.

If the parent/guardian agrees to take part, copies of the signed forms will be made, and one given to the parents.

Participants will be informed that they can withdraw from the study at any time without an explanation and their child’s care will not be affected.

### Randomisation

Once the consent is sought and the forms completed, the participant data will be entered on the central online randomisation system and the participant will be randomised using a secure online randomisation system to either the pregraft orthodontic group or no orthodontic treatment group, in a 1:1 ratio. The allocation sequence generation will be generated through the randomisation programme. The randomisation will be stratified on gender to get an equal number of males and females. The participants will be allocated by an independent researcher blinded to all the study data except to patient gender. Each participant will then be assigned a unique randomisation number.

### Interventions

Orthodontic treatment will be provided for the treatment group. The treatment will involve fixed braces for the top teeth with or without expanders to widen the top jaw. The brace technique will be a clinical judgement and will be done in real-world setting. The treatment with a fixed brace can take up to 15 months. This will be followed by alveolar bone graft surgery to repair the cleft palate. The alveolar bone graft surgery will be standardised across all surgeons.

In the control group, no orthodontic treatment will be provided but the patients will have alveolar bone graft surgery.

The trial will be carried out in a real-world setting and the patient will not be prohibited from any other care during the trial.

### Blinding

Due to the nature of the intervention, it is not possible to blind the investigator or the patient. The outcome assessor, however, will be blinded to the allocated group.

### Outcome measures

The primary outcome will be:

- Success of alveolar bone graft assessed from CBCT and oblique occlusal (Kindelan method)

The secondary outcome measures will be:

- Quality of the dento-occlusal outcome measured on study models using 10-year-old index scores
- Qualitative assessment using the validated quality of life questionnaires
- Breakages
- Quality-adjusted cost analysis

The operator will record a patient as non-compliant if:

- Subject refuses to wear the appliance
- Breakage of appliance for more than 3 times
- Measurement indicates a lack of clinical progress after 9 months of appliance wear

### Data collection

Data will be collected at the following points:

- ▪ T1: At the start of treatment/baseline
- ▪ T2: 9 months from baseline
- ▪ T3: Pre alveolar bone graft
- ▪ T4: 3 months post alveolar bone graft
- ▪ T5: 6 months post alveolar bone graft

Records, i.e., study models, intra-oral and extra-oral photographs and a oblique occlusal radiograph will be taken at each time point. A CBCT will be taken at T5 stage.

In addition, the following data will be collected from patients’ notes:

- Number of attendances, including failed and cancelled appointments
- Overall treatment duration
- Frequency of appliance breakages, if any
- Any other emergencies

Patients will be required to fill in the Quality of Life questionnaires (CPQ_8-10_) which will inform us how the appliances will affect them as part of the qualitative assessment. The CPQ_8-10_ is a validated questionnaire and has been used in other orthodontic trials.

### Data collection and management

All trial data will be recorded on case report forms (CRF). The forms from the trial centre will be securely transported to the data coordinating centre. They will be entered into a trial specific database by the Trial Manager at the data coordinating centre.

#### Radiographs

The oblique occlusal radiographs will be taken at four time points; start of treatment (T1), prealveolar bone graft surgery (T3), 3 months post surgery (T4), and 6 months post surgery (T5).

The CBCT radiograph will be taken at the final data collection point, 6 months post surgery (T5).

The radiographs will be saved with a unique patient ID and transported to the data coordinating centre using an encrypted USBs. They will be securely stored onto a trial-specific computer within the data coordinating centre.

**Fig. 2:**
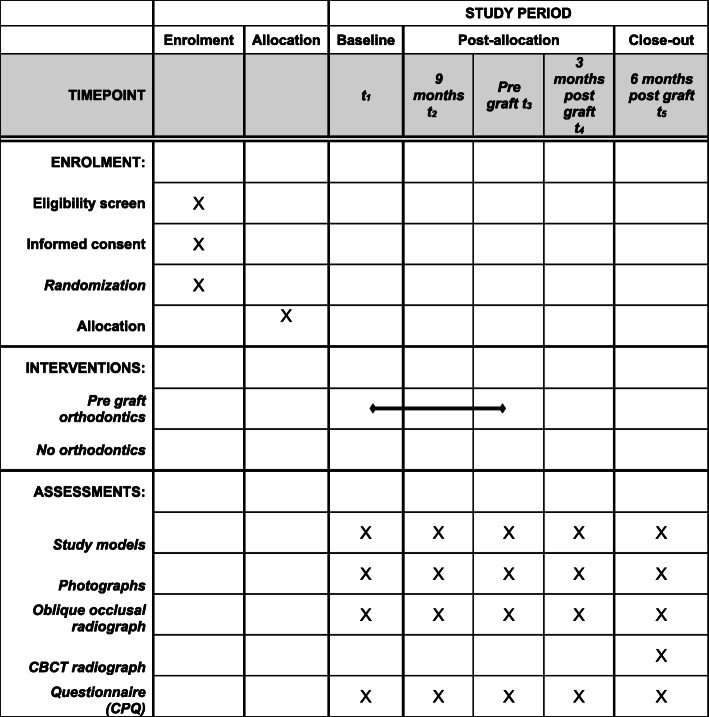
SPIRIT checklist for the schedule of enrolment, interventions and assessments

The SPIRIT checklist for the schedule of enrolment, interventions, and assessments is shown in Fig. 2.

### Study models

The upper and lower impressions along with a wax bite of occlusion which would be obtained at all time points and will be casted with dental stone at the trial sites. The models will then be transported to the data coordinating centre and stored in a secure site.

### Photographs

A set of seven extraoral and eight intraoral photographs will be obtained at all time points. The photographs will be saved using a unique study ID on an encrypted USB and transported to the data coordinating centre.

### Data analysis

#### Sample size estimation

The sample size was based on showing a difference between groups for the primary outcome, success as defined by a Grade 1 on the Kindelan bone-fill index. Based on previous research (Paterson et al.), it is estimated that 60% of the treated will have a successful outcome. It is estimated that only 25% of the untreated group will have a successful outcome. With a 5% significance level and 80% power, it is calculated that 31 subjects per group, 62 in total are required for the study. To allow for a 10% non-compliance rate, 72 subjects will be recruited into the study.

#### Proposed method to ensure the reliability of measurements

Twenty oblique occlusal radiographs will be evaluated with Kindelan index, a week apart to ensure good reliability of the measurement. The Error will be evaluated with the paired *t* test, Bland and Altmann and intra-class correlation coefficient to test both systemic and random errors.

The trial will be conducted in line with Declaration of Helsinki (1964) and reported using the Consolidated Standard of Reporting Trials (CONSORT).

### Data analysis

Data will be analysed by an independent statistician. A detailed analysis will be planned before the final analysis of the trial. The main features of the statistical plan are mentioned here.

Data analysis will be carried out at the end of the study.

The primary outcome, Kindelan bone-fill index, is an ordinal measure and will be compared between groups using the Mann-Whitney test. Additionally, the outcome will be categorised as successful (grade 1) or unsuccessful (grades 2–4). The chi-square test will be used to compare the categorised outcome between groups.

Independent sample *t* test and 95% confidence intervals will be used for all continuous outcomes, for comparison between the groups. The dichotomous outcomes will be compared using chi-square test and the effect estimates will be reported using relative risk and 95% confidence interval. The qualitative outcomes will be compared using chi-square test.

A multivariable linear regression analysis will be carried out to assess the effect of pre-treatment complexity, gender and orthodontic treatment on the overall quality of alveolar bone graft success. The primary analysis will be on an intention-to-treat principle; the results of all subjects will be accounted for, regardless of the outcome of the treatment.

### Protocol deviations

- ▪ All patients failing an appointment will be sent another.
- ▪ Those wishing to withdraw from the trial may do so at any point with no detriment to their continuing treatment. When a patient withdraws from the study the relevant records will be taken at the point of withdrawal.
- ▪ Appliance breakage—every effort will be made for the patient to be seen by a principal operator. However, in certain circumstances, the patient may be seen by a second investigator.
- ▪ If there are more than four breakages for any one patient, then the patient will be removed from the study and records taken at the final breakage appointment.

### Data access

The investigators and the investigators’ designated staff personnel will have access to their centre’s data. All data collected will be analysed by an independent statistician. Subject identifiable data will remain confidential. Access to subject data and research data will be restricted per applicable data protection laws/regulations and research requirements.

### Protocol compliance

Accidental protocol deviations can happen at any time. They will be adequately documented on the relevant forms and reported to the Chief Investigator immediately.

Deviations from the protocol which are found to frequently recur are not acceptable and will require immediate action, as may be defined as serious breaches.

All potential protocol deviations/violations will be reported in line with institutional requirements.

### Recruitment strategy

A dedicated clinical recruiter will be available at each site to identify potential participants and alert the investigators. Additionally, the clinical recruiter will also have access to the database to identify patients requiring an alveolar bone graft. Written leaflets with inclusion criteria will be put up on the clinic waiting room educating patients on the clinical trial.

### Confidentiality

All CRFs will have a unique patient ID allocated upon randomisation. The DCC will preserve the confidentiality of the participants taking part in the trial. The Indian Data Protection Law 2019 and all ICMR guidelines will be followed in this trial.

### Trial monitoring

The study will be overseen by a Study Advisory Group with nominations from the Cleft Lip and Palate Society in India. This will include the members of the study team in addition to an independent chairperson and independent experts in cleft orthodontics, cleft surgery and biostatistics. We will also include patient representatives from Sree Balaji Dental College, BIHER. The Study Advisory Group will have the following specific roles (i) regularly provide advice into the study, (ii) advise on key parameters to explore the Value of Information analysis, and (iii) advise on the qualitative component to enable parents and patients to voice their perspectives and priorities.

### Data and Safety Monitoring Board (DSMB)

The DSMB will comprise of a clinician, a statistician and a layperson. The DSMB will be responsible for reviewing and assessing recruitment, interim monitoring of safety, trial conduct and external data.

### Ethics

The protocol has gained a favourable opinion from the Bharath Institute of Higher Education and Research (SBDCH/IEC/12/2019/2). Current trial status and timeline: The ethical approval was obtained on 18th December 2019. The trial recruitment will commence from 1st November 2020. The recruitment is planned for 12 months and the data collection is likely to last for 24 months from the last recruited patient.

### Ancillary and post-trial care

This will be in accordance with the Indian Council of Medical research guidelines.

insurance would be covering all patients participating in the trial for trial-related harms.

The study is a real-world study and is carried out as we would do for patients routinely seen in clinical care. The care for these patients post trial will continue as it would for patients seen routinely in the clinic.

### Dissemination

The trial principal investigator will publish the results of the study in a peer-reviewed scientific journal and presented at the national and international conference. The results from the proposed study will be used for discussions with stakeholders and policymakers. Support will be requested from both for communicating the study results, and any clinical guidance that stems out from this project to the general public.

## Discussion

The PABO study has the goal of reducing the burden of care for patients with cleft palate. These children undergo treatment from different specialists at various timepoints in their life. Orthodontic treatment is a lengthy course of treatment and to add this in addition to other orthodontic treatments in the later part of their lives along with the already increased burden of care from other problems must be justified. This early orthodontic treatment is mainly carried out to make the alveolar bone graft surgery less complicated and to a minimal extent, align the front teeth for aesthetic reasons. However, if this can be avoided with no compromise on the quality of overall outcome at the of surgery will be a huge benefit for these patients. This study will allow us to explore the effectiveness of pre graft orthodontic treatment and inform both clinicians and patients on the most effective way of treatment for children with unilateral cleft palate.

## Trial status

Recruitment start date 01/08/2021. Estimated completion date: 01/08/2022.

Protocol version number 3.3.

## Data Availability

The datasets from the study will be available from the corresponding author on request.

## Abbreviations

CRF: Case report forms
DCC: Data coordinating centre
DSMB: Data and Safety Monitoring Board
UCLP: Unilateral cleft lip and palate
CPQ: Child Perception Questionnaire
CBCT: Cone beam computed tomography
